# Optimized Glycopeptide Enrichment Method–It Is All About the Sauce

**DOI:** 10.1021/acs.analchem.2c00524

**Published:** 2022-06-28

**Authors:** David Morgenstern, Hila Wolf-Levy, Nili Tickotsky-Moskovitz, Itzik Cooper, Aron S. Buchman, David A. Bennett, Michal Schnaider Beeri, Yishai Levin

**Affiliations:** † The de Botton Institute for Protein Profiling, Nancy and Stephen Grand Israel National Center for Personalized Medicine, Weizmann Institute of Science, Rehovot 7610001, Israel; ‡ Icahn School of Medicine at Mount Sinai, Department of Psychiatry, New York, New York 10029, United States; § The Jospeh Sagol Neuroscience Center, Sheba Medical Center, Tel Hashomer 52621, Israel; ∥ Rush Alzheimer’s Research Center, Rush University, Chicago, Illinois 60612, United States

## Abstract

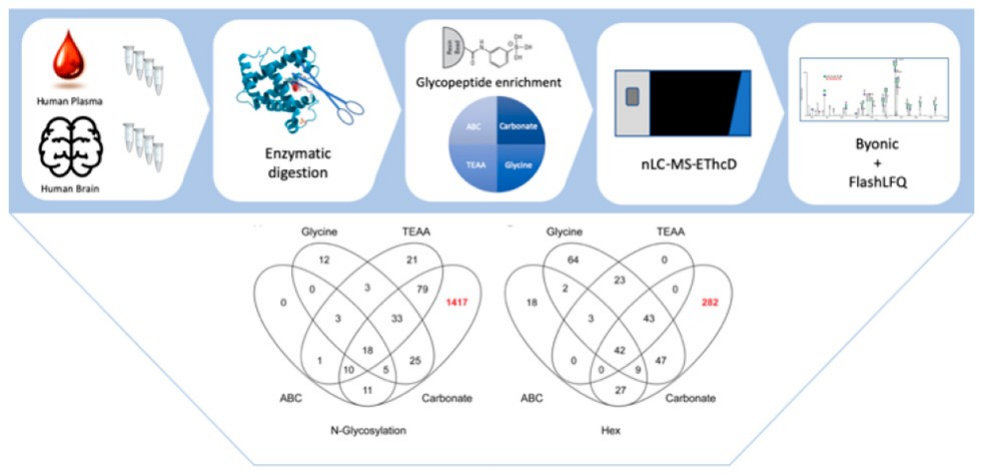

Protein glycosylation is a family of posttranslational modifications that play a crucial role in many biological pathways and diseases. The enrichment and analysis of such a diverse family of modifications are very challenging because of the number of possible glycan–peptide combinations. Among the methods used for the enrichment of glycopeptides, boronic acid never lived up to its promise. While most studies focused on improving the affinity of the boronic acids to the sugars, we discovered that the buffer choice is just as important for successful enrichment if not more so. We show that an amine-less buffer allows for the best glycoproteomic coverage, in human plasma and brain specimens, improving total quantified glycopeptides by over 10-fold, and reaching 1598 N-linked glycopeptides in the brain and 737 in nondepleted plasma. We speculate that amines compete with the glycans for boronic acid binding, and therefore the elimination of them improved the method significantly.

## Introduction

Protein glycosylation is an important posttranslational modification that affects a myriad of biological processes, including interaction with dedicated proteins, promotion of protein stability and folding, resistance to proteolytic cleavage, and microenvironment generation and maintenance. In turn, these varied protein modifications affect cellular adhesion, immune response, protein–receptor interaction, protein transport, and secretion and cellular infection by pathogens.^1^ Lastly, glycoproteins are a significant part of FDA-approved cancer biomarkers.^2^

Unlike other posttranslational modifications (PTM), protein glycosylation encompasses highly diverse glycan compositions and structures, resulting in hundreds of different structures and compositions. While it is estimated that a very large fraction of the proteins are glycosylated (30%–50%),^3^ it is a much smaller fraction of the total proteotypic peptides in bottom-up proteomics experiments, estimated at 3%. Thus, an in-depth survey of glycosylated peptides in a sample by mass spectrometry-based proteomics requires an enrichment step that will remove the nonglycosylated peptides. However, the immense complexity of glycopeptides represents a significant biochemical challenge, simply because of the breadth of physiochemical characteristics of both glycans and the peptides carrying them.

While many glycopeptide enrichment strategies have been developed (see review (4)), in most cases either the enrichment efficiency is not very high or they show selectivity toward subsets of the glycopeptides, thereby introducing significant bias to experimental efforts to profile them. The most common methods currently are hydrophilic interaction liquid chromatography (HILIC) and the use of multiple lectins.^4,5^ HILIC is a very popular method of enrichment as it is both straightforward and simple to use. However, its enrichment efficiency is low (as it enriches only very hydrophilic peptides), and it is biased against hydrophobic glycopeptides as well as peptides with small glycans. Lectin affinity provides a targeted approach for the enrichment of specific glycan subclasses.^4^ The use of multiple lectins allows the expansion of the targeted glycan population, but it does not achieve unbiased enrichment.

Enrichment using boronic acid may be the most promising method for glycopeptide enrichment. Boronic acids allow reversible, pH-dependent covalent binding to cis-diols that can be found in sugars. They have the potential to produce unbiased enrichment of the total glycopeptide population in the proteome, but unfortunately it is also the most underperforming enrichment method so far. There are many method papers that describe novel chemistries and approaches for the use of boronic acids for glycopeptide enrichment. While some experiments yielded high glycoproteomic coverage, they did require prefractionation to achieve this.^6−9^

We optimized the boronic acid-based protocol by trying various buffers during the enrichment procedure, which led to a significant improvement in the number of identified and quantified glycopeptides.

## Materials and Methods

Human plasma (cat. number p9523), branched poly(ethylene imine) (PEI 25 kDa), glycine, ammonium bicarbonate (ABC), triethylamine, sodium carbonate, sodium bicarbonate, potassium chloride, 4-(4,6-dimethoxy-1,3,5-triazin-2-yl)-4-methylmorpholinium chloride (DMTMM), and AF-Tresyl-650 M beads were obtained from Sigma-Aldrich, 6-carboxyebenzoboroxole from Santa-Cruz Biotechnology, methanol from J. T. Baker, and acetonitrile from Biolab, Israel. Empty TopTips were obtained from GlyGen.

Human frozen, postmortem brain tissue was obtained from the Rush Memory and Aging study,^10^ whose main goal is to identify the postmortem indices linking genetic and environmental risk factors to the development of Alzheimer’s disease (AD). Methods for human brain tissue harvesting are detailed in ref (10). The study received an institutional review board approval.^10^

### Preparation of Polyethylenimine (PEI)–Benzoboroxole Beads

One gram of AF-Tresyl-650 M beads was washed with methanol three times and derivatized (under the assumption of 5 μmol/g binding capacity) with 5 mL of 50 mM PEI in PBS at room temperature (RT) for 12 h in a 30 rpm rotation. Beads were washed with methanol five times to dehydrate the beads and remove both reagent and buffer. Assuming 600 amines per 1 molecule of PEI, 5 μmol PEI translates into 3 mmol amines bound to the beads. The beads were further functionalized with 5-benzoboroxole by adding 3 mL of 1 M DMTMM and 1 M 5-benzoboroxole in methanol for 16 h at RT in rotation. Beads were washed with methanol five times to remove excess reagent and washed three times with 20% ethanol for storage. Beads were stored at 20 μg/μL beads in 20% ethanol.

### Brain Sample Lysis

Brain tissue was transferred to 2 mL bead-beating tubes (Precellys lysing kit p000918-LYSK0-A), and 500 μL of 5% SDS in 50 mM Tris buffer was added to the tubes. The samples were homogenized using a Bead Beater (PRECELLYS Evolution, Bertin Technologies) for 10 s at 10 000 rpm, 6 s pause, and 1 min on ice, three times. Then three cycles of 20 s, 6800 rpm, and 30 s pause between cycles were carried out. The tubes were then transferred to a benchtop centrifuge at 13 000*g* for 10 min at 4 °C. The supernatant fluid was transferred to new Eppendorf tubes and frozen at −80 °C.

Plasma and brain samples were mixed vol:vol with 10% SDS, 50 mM Tris, pH 7.55, for a total volume of 50 μL. The samples were heated for 15 min at 96 °C with 500 rpm rotation and then sonicated for 10 cycles (Bioraptor Pico, Diagnode) and centrifuged for 8 min at 13 000*g*.

### S-Trap Digestion

For all samples, the total protein concentration was measured using a BCA assay. One hundred micrograms of each sample was used for downstream preparation. Dithiothreitol (DTT) was prepared fresh in 50 mM ammonium bicarbonate and added to a final concentration of 5 mM. Samples were then incubated at 56 °C for 1 h. Iodoacetamide was prepared fresh in 50 mM ammonium bicarbonate and added to a final concentration of 10 mM. Samples were incubated in the dark for 45 min. Phosphoric acid was then added to the samples to a final concentration of 1%. The samples were mixed with 350 μL of 90% MeOH + 10% 50 mM ammonium bicarbonate and then transferred to the S-trap cartridge (Protifi, USA), centrifuged for 1 min at 4000*g*, washed three times with 400 μL of 90% MeOH + 10% 50 mM ammonium bicarbonate, and then centrifuged at 4000*g* for 1 min. Four microliters of 0.5 μg/μL trypsin in 125 μL in ammonium bicarbonate (50:1 protein amount:trypsin) was added to the samples. Samples were incubated at 37 °C overnight. The next day, peptides were eluted using 80 μL of 50 mM ammonium bicarbonate, which was added to the S-trap cartridge and centrifuged at 4000*g* for 1 min into new tubes, and the peptides were then collected. Then, a second digestion was performed using 4 μL of 0.5 μg/μL trypsin in 50 mM ammonium bicarbonate, which was added to the eluted samples and incubated at 37 °C for 4 h. Two more elations from the S-trap cartridge were performed: one was carried out with 80 μL of 0.2% formic acid, which was added to the S-trap cartridge and spun down at 4000*g* for 1 min. The second was done using 80 μL of 50% acetonitrile + 0.2% formic acid, which was added to the cartridge and spun down at 4000*g* for 1 min. The three elutions were mixed and dried using a vacuum centrifuge (Centrivac, LabConco).

### Glycosylation Enrichment

We compared four buffers for the enrichment step, as shown in Table 1.

**Table 1:** List of the Four Different Buffers Used for the Enrichment Step

	loading buffer	wash buffer
glycine	50% MeCN, 0.5 M glycine pH 10.5	50% MeCN, 0.5 M glycine pH 10.5
ABC	50% MeCN, 0.5 M ABC pH 8.5	50% MeCN, 0.5 M ABC pH 8.5
TEAA	50% MeCN, 0.5 M TEAA pH 10.5	50% MeCN, 0.5 M TEAA pH 10.5
carbonate–bicarbonate	50% MeCN, 50 mM carbonate pH 10.5, 1 M KCl	50% MeCN, 50 mM carbonate pH 10.5

Twenty microliters of derivatized beads (per sample) were spun down to remove storage buffers and washed with the loading buffer twice. Samples were dissolved in 50 μL of loading buffer and added to the beads. Beads were incubated in rotation at RT for 30 min, and the beads were then loaded on empty TopTips (10 μL) and spun in a centrifuge at 376*g* for 30 s to remove the solution. Beads were washed twice with the loading buffer and twice with the washing buffer. Twenty microliters of 5% formic acid/50%ACN was added to the samples, incubated for 10 min at RT to elute the bound glycopeptides. The samples were spun for 60 s. The above volume of elution buffer was added again and eluted immediately.

#### Mass Spectrometry

Samples were reconstituted in 15 μL of 3% acetonitrile/0.1% formic acid. Samples were loaded on a Symmetry trap column (C18, 180 μm × 20 mm, 5 μm, 100A, Waters Inc.), resolved using a HSS T3 analytical column (C18, 75 μm × 250 mm, 1.8 μm, 100A, Waters Inc.), and mounted on a nanoAcquity instrument running at a flow of 0.35 μL/min using a gradient of 4–25% for 125 min followed by 25–40% for 30 min. Data was acquired on an Orbitrap Fusion Lumos instrument (Thermo Fisher Scientific) running at a 3 s top-speed DDA method. MS1 scans were performed at 120 000 resolution (@200 *m*/*z*) in the 400–1800 *m*/*z* range. The most abundant ions at charge states 2–8 and at minimum 5 × 10^4^ intensity were chosen for fragmentation. Precursors were isolated in the quadrupole using a 1 *m*/*z* isolation window, and MS2 fragmentation was performed using EThcD using calibrated charge-dependent parameters with supplemental activation of 15 NCE. MS2 data was acquired at 15 000 resolution (@200 *m*/*z*) using a first mass of 120 *m*/*z* with standard AGC and maximum injection time of 120 ms.

#### Data Analysis

Data was searched using the Byonic search engine^11^ against the human proteome (SwissProt Dec 20) with Byonic’s common contaminants library appended and against an 84 plasma glycan library for blood and 182 glycan library for the brain samples (supplied by Byonic). Searches were performed using specific cleavage of trypin with two missed cleavages allowed, with EThcD fragmentation. Mass tolerances were set to 10 ppm for MS1 and 20 ppm for MS2. The following modifications were allowed: fixed carbamidomethylation on C, variable oxidation on M (common 1), deamidation on NQ (common 1), phosphorylation on STY (rare 1), hex on K (common 2), protein N-terminal acetylation (rare 1), and peptide N-terminal pyroGlu (rare 1), for a total of two common modifications and one rare modification.

Identifications were filtered for a Byonic identification score >150 and manually inspected identifications in the score range 150–300.

Glycopeptides were quantified using FlashLFQ^12^ standalone GUI version using default settings with normalization and “match between runs” enabled.

We further filtered the quantitative data for a minimum three valid values out of four replicates, in at least one group.

The mass spectrometry proteomics data has been deposited to the ProteomeXchange Consortium via the PRIDE^13^ partner repository with the data set identifier PXD031177 and 10.6019/PXD031177.

## Results

We compared four different buffers for use with boronic acid-based enrichment of glycopeptides, which are commonly used in glycoproteomics: three at pH 10.5 and one at pH 8.5, based on the study showing improved binding properties at less alkaline pH.^9^ The three buffers were triethylammonium acetate pH 10.5 (a tertiary amine, TEAA), glycine (primary amine), and carbonate–bicarbonate (nonamine). At pH 8.5 we used ammonium bicarbonate (ABC).

The comparison was performed using four identical aliquots of human plasma and four identical replicates of human brain samples. This enabled testing of the different buffers as well as quantitative reproducibility of each method.

**Figure 1:**
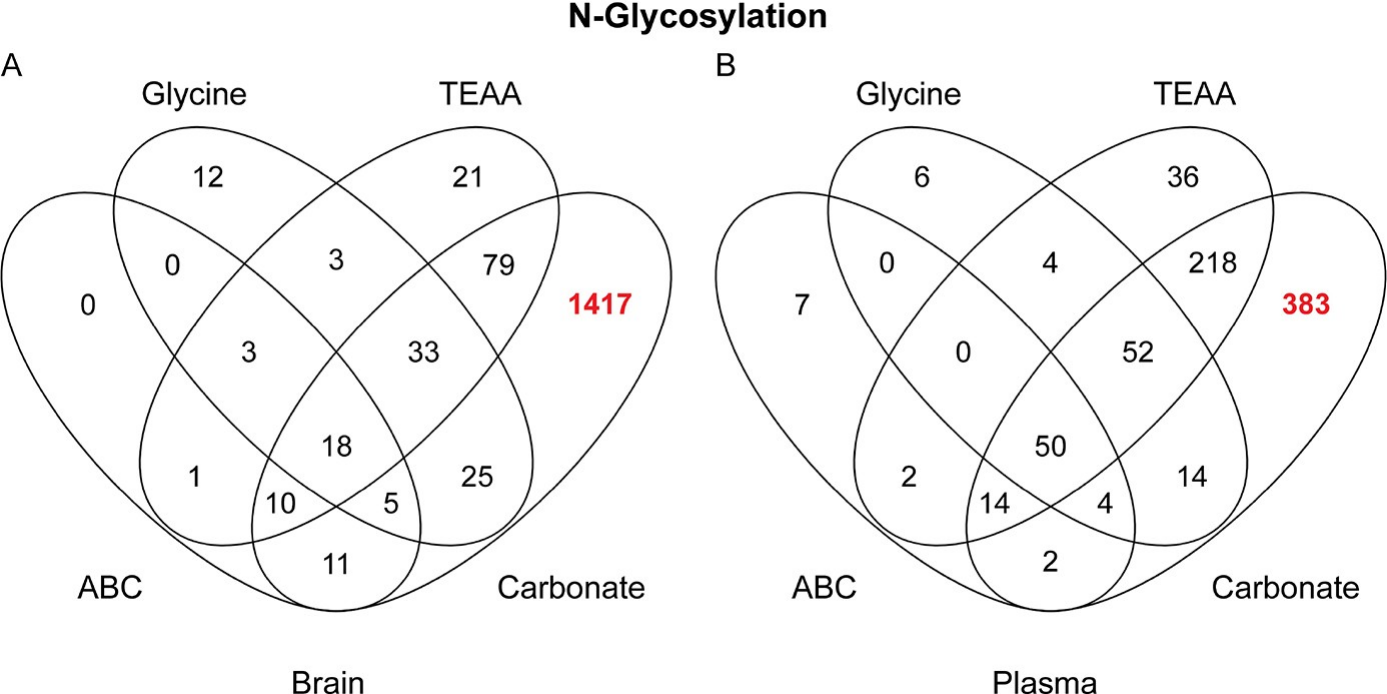
Venn diagrams comparing the number of quantified N-linked glycopeptides (with at least three valid values) using different buffers for enrichment: (A) brain and (B) plasma.

First, we checked the overlap in quantified glycopeptides, comparing each method, separately for N-linked glycopeptides and glycated peptides (Hex modification). We consider quantified glycopeptides as those having at least three intensity values out of the four replicates per condition. Figure 1 shows a comparison of the identified N-glycopeptides between the different buffers for brain and plasma. It can be seen that the carbonate buffer significantly outperforms the other buffers in both sample types, with a total of 1598 N-linked glycopeptides in the brain, which is 10 times higher than the next best buffer. In the plasma samples, we quantified a total of 737 N-linked glycopeptides. The reason for the higher coverage in tissue is most likely due to the extreme dynamic range of protein abundance in nondepleted plasma.

**Figure 2:**
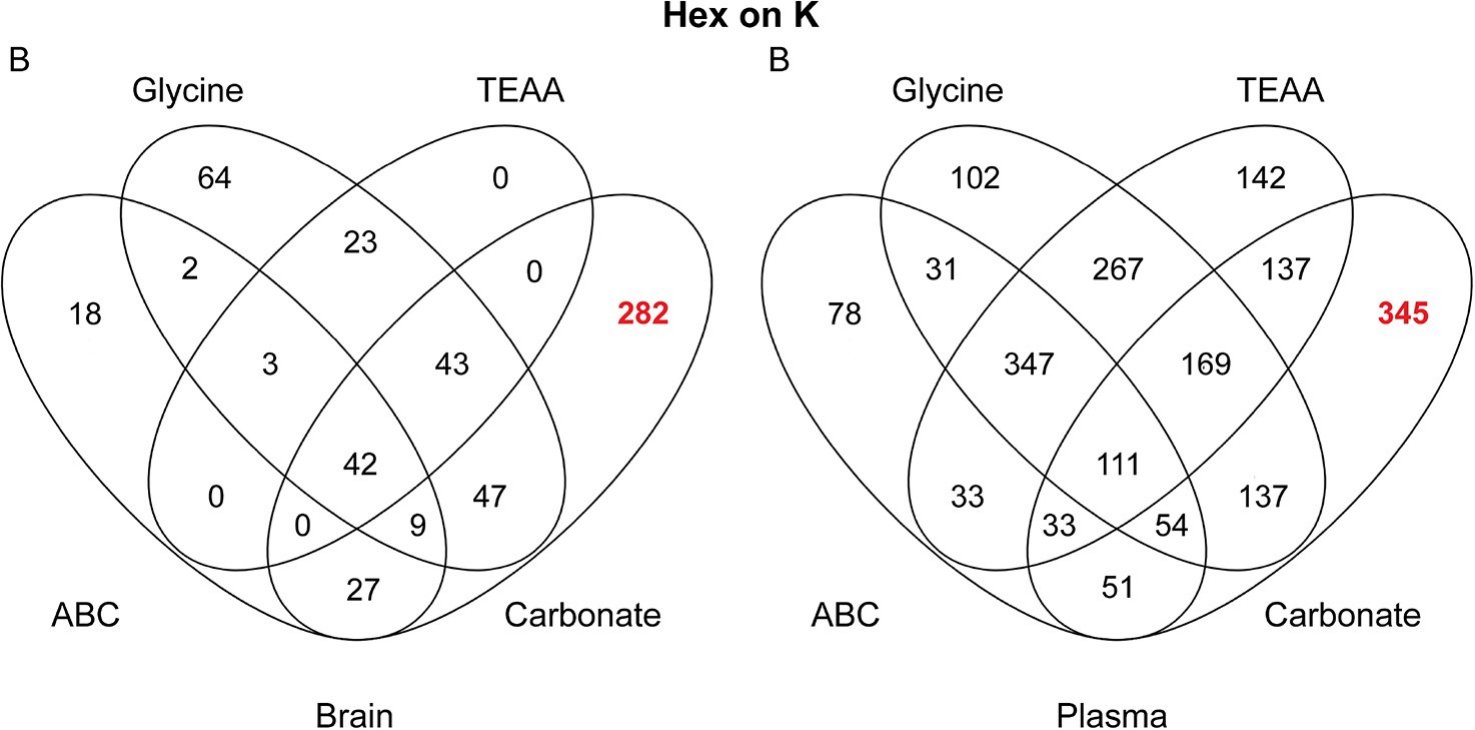
Venn diagrams comparing the number of quantified glycated peptides (Hex modification, at least three valid values) using different buffers for enrichment: (A) plasma and (B) brain.

The difference between carbonate and the other buffers was less striking when comparing glycated peptides with the small Hex modification in plasma but is much more obvious in the brain samples (Figure 2).

**Figure 3:**
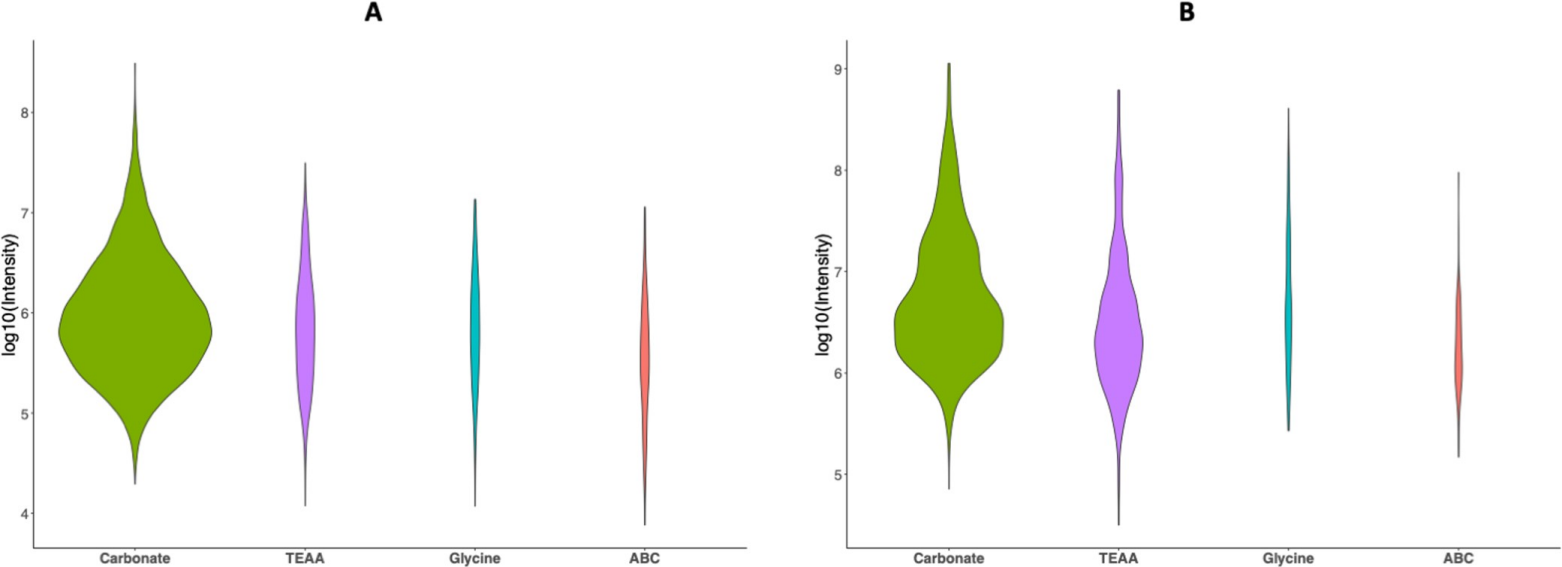
Violin plots comparing quantified N-linked glycopeptides for each sample type. The carbonate buffer outperforms all others, except for the Hex-modified peptides in the plasma samples. The figure shows density plots based on the relative peak height of the glycopeptides.

Next, we compared the quantified N-linked glycopeptides for each method and each sample type. As shown in Figure 3, the carbonate buffer significantly outperforms the others. This is particularly apparent for low-intensity glycopeptides.

**Figure 4:**
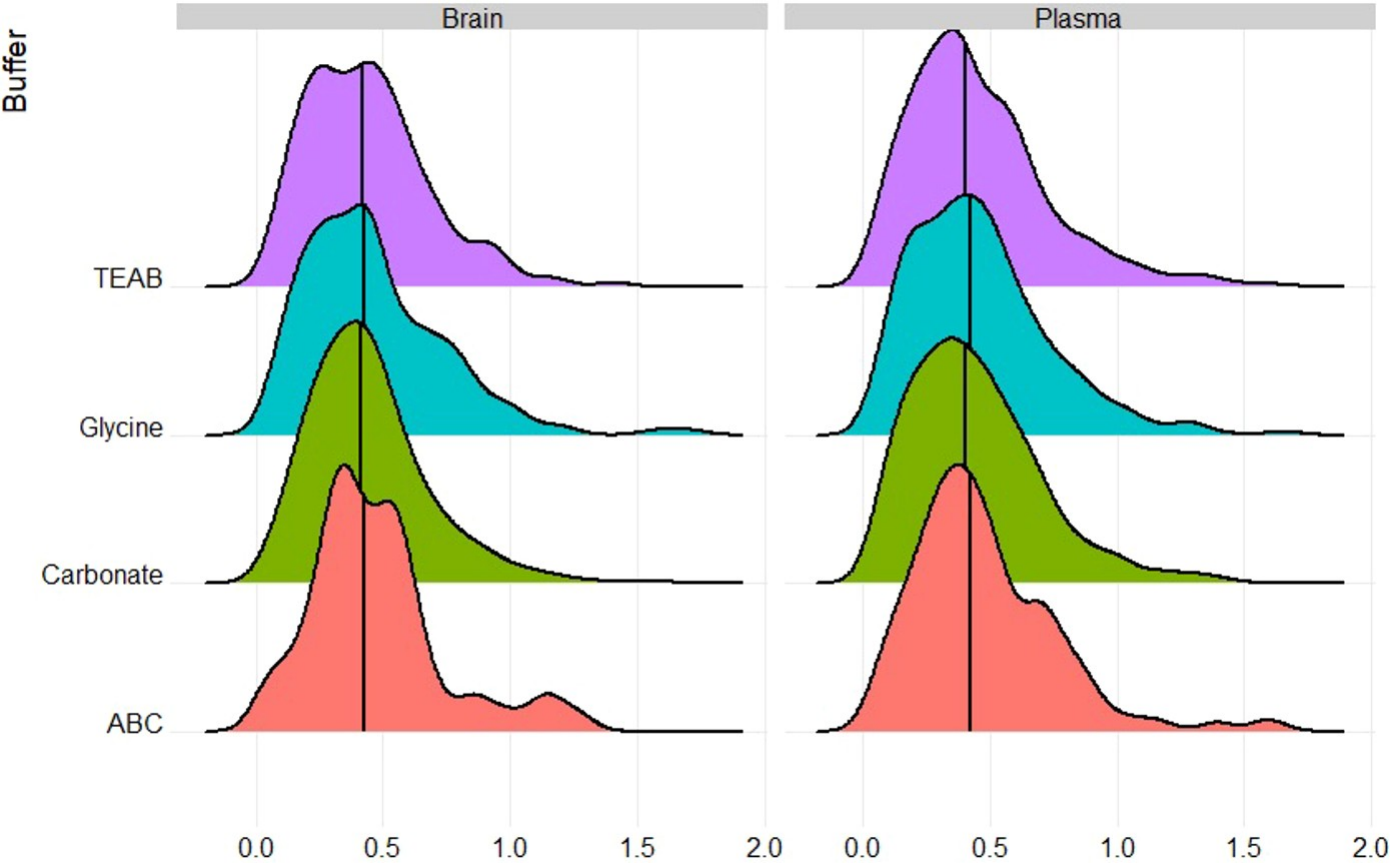
Density plots of the intensity coefficient of variation (CV, standard deviation divided by the mean) based on the replicates of each buffer for the plasma and brain samples. The vertical lines show the median. Plasma: ABC, 0.42; carbonate, 0.40; glycine, 0.42; TEAA, 0.40. Brain: ABC, 0.43; carbonate, 0.41; glycine, 0.43; TEAA, 0.42.

Finally, we used the glycopeptide intensity measurements, based on peptide peak height, for quantitative evaluation of each method. Figure 4 shows density plots of the distribution of the coefficient of variation (CV, standard deviation divided by the mean) based on the replicates of each buffer. This data was generated by the FlashLFQ tool as described in the Materials and Methods section. It can be seen that the median CVs were around 0.4, which is quite good for peptide level analysis.^14^ This means we would be able to reliably measure biological changes in real life samples.

## Discussion

We found that boronic acid represents the best option for the unbiased enrichment of glycopeptides. The vast diversity of glycans and the peptides that carry them makes their identification extremely difficult. The most common methods (HILIC and multilectin affinity) still suffer from the biases ingrained in their methods of enrichment.^15−17^ In this respect, the nonspecific nature of boronic acid binding to 1,2- and 1,3-diols has the potential for an unbiased enrichment of glycopeptides. Unfortunately, so far most of the publications investigating boronic acids as means of glycopeptide enrichment failed to translate into widespread use in biological studies.

Looking at the physiochemical characteristics of boronic acids and their support,^18^ we concluded that we will have to overcome several molecular interactions that contribute to nonspecific binding of nonglycosylated peptides. We therefore included a high-salt buffer to suppress electrostatic and hydrogen interactions and at least 50% MeCN to overcome hydrophobic interactions with the boronic acid support. Most existing protocols include amine-based buffer,^9,19,20^ which is counterintuitive as boronic acids are known to interact with amine-based chromatography supports^21^ and are used to catalyze amine–carboxyl reactions.^22^ Combined with the low binding coefficient of boronic acid and the relative abundance of buffer amines versus glycosylated peptides, we believe that amines can displace diols from the binding sites.^9^ Thus, we investigated the use of amine and nonamine buffers in alkaline pH and compared that to less alkaline pH.

The results shown here suggest that there is a certain bias common to all buffers for glycated peptides over glycosylated peptides. Glycation enrichment was successful with any buffer in highly alkaline pH, while N-glycosylation was heavily dependent on a specific buffer composition, i.e., amineless buffer. This is suggestive of a certain preference toward binding fructose or glucose, which are the most common saccharides involved in glycation, compared to monosaccharides that occur in N- and O-glycosylation. Using a carbonate buffer, we minimized nonspecific binding of amines with the boronic acid moieties by reducing competition for the glycopeptides. This differential preference may be used either to purify only glycated peptides or to remove them in an initial step retaining only glycosylated peptides.

In summary, we showed that enrichment buffer optimization overcame nonspecific interactions of the boronic acid with buffer molecules, resulting in a significant improvement of glycoproteomic coverage in highly complex biological samples, plasma and brain tissue. The improvement in enrichment efficiency will allow researchers to capitalize on glycoproteomics research on invaluable clinical samples to investigate the role of glycopeptides in health and disease.
